# Draft Genome Sequence of Strain BF-4, a *Lysinibacillus*-Like Bacillus Isolated during an Anthrax Outbreak in Bavaria

**DOI:** 10.1128/genomeA.00918-14

**Published:** 2014-10-09

**Authors:** Markus Antwerpen, Enrico Georgi, Pia Zimmermann, Stefan Hoermansdorfer, Hermann Meyer, Gregor Grass

**Affiliations:** a Bundeswehr Institute of Microbiology, DZIF, Partner Site of German Center for Infection Research, Munich, Germany; b Bavarian Agency for Health and Food Safety, Oberschleissheim, Germany

## Abstract

We report the draft genome sequence of *Lysinibacillus* sp. strain BF-4. Strain BF-4 has a notably small genome for a free-living bacillus, with a size of 2.63 Mbp. In agreement with phenotypic observations, the genome lacks genes essential for endospore formation.

## GENOME ANNOUNCEMENT

Little is known about the accompanying bacterial flora in anthrax-infected animals. In the summer of 2009, a herd of cattle was infected in upper Bavaria by *Bacillus anthracis*, the causative agent of anthrax. Four cows succumbed to the disease, and from their carcasses either DNA or *B. anthracis* was isolated (1). Additional Gram-positive rods were identified and further analyzed from one spleen that was culture negative but DNA positive for *B. anthracis*. A non-endospore-forming isolate (named BF-4) most closely related to the genus *Lysinibacillus* (2) was cultured. This strain exhibited growth characteristics similar to *Bacillus cereus sensu lato* strains. Similar to *B. anthracis*, isolate BF-4 was susceptible to penicillin and nonhemolytic. Partial sequencing of the 16S rRNA gene revealed 99% identity to a yet-uncultured *Bacillus* sp., isolated from the rumen of a yak (*Bos mutus*) (FJ172860.1).

Whole-genome shotgun (WGS) sequencing of strain BF-4 was performed using Ion Torrent personal genome machine technology. For the WGS library, 2,537,806 reads with a total of 489,277,968 bp were generated. About 99.4% of the reads were assembled into 180 contigs using Newbler version 2.6 (Roche) to reach 92.8-fold coverage. The assembled contigs were submitted to the RAST annotation server for subsystem classification and functional annotation (3). Coding sequences (CDSs) were assigned using BLASTp with KEGG orthology (KO) (4). The G+C content was calculated using an in-house Python script.

The total length of the draft genome shotgun sequence of strain BF-4 was 2,626,059 bp and the mean G+C content was 40.2%. The chromosomal sequence comprised 118 large contigs (>500 bp) with 87.3-fold coverage. The 16S rRNA gene sequence revealed 97% identity to *Lysinibacillus* (*Bacillus*) *sphaericus* C3-41 (GenBank accession no. NC_010382.1) (5), the closest relative with a published genome sequence to date. Blast analysis of large contigs of BF-4 showed a similarity of only 75% as the best hit, with only partial sequence alignments between the two organisms. Isolate BF-4 presents notable features uncommon for members of the *Bacillaceae*, such as a remarkably small genome size and the conspicuous absence of most genes required for endospore development. These properties make this bacterium, which is easy to grow *in vitro*, outstanding and an ideal subject for further studies on the poorly characterized accompanying microbial flora coisolated during anthrax disease outbreaks.

### Nucleotide sequence accession numbers.

The draft genome sequence for strain BF-4 has been included in the GenBank WGS database under the accession number JPUW00000000. The version described in this paper is the ﬁrst version, JPUW10000000.
